# Efficacy of a Smart Antisnore Pillow in Patients with Obstructive Sleep Apnea Syndrome

**DOI:** 10.1155/2021/8824011

**Published:** 2021-01-14

**Authors:** Tsung-Te Chung, Ming-Tsung Lee, Ming-Chou Ku, Kai-Chieh Yang, Cheng-Yu Wei

**Affiliations:** ^1^Department of Otolaryngology, Show Chwan Memorial Hospital, Changhua 500, Taiwan; ^2^Sleep Center, Chang Bing Show Chwan Memorial Hospital, Changhua 505, Taiwan; ^3^Research Assistant Center, Show Chwan Memorial Hospital, Changhua 500, Taiwan; ^4^Department of Nursing, Hungkuang University, Taichung 433, Taiwan; ^5^Department of Orthopedics, Show Chwan Memorial Hospital, Changhua 500, Taiwan; ^6^Department of Exercise and Health Promotion, College of Kinesiology and Health, Chinese Culture University, Taipei 111, Taiwan

## Abstract

**Objective:**

Untreated obstructive sleep apnea syndrome (OSAS) increases the risk of cardiovascular, dementia, and motor vehicle accident events. However, continuous positive airway pressure (CPAP) which is the gold standard treatment is not acceptable for many patients with OSAS. Development of devices for the patients of nonadherence to CPAP is necessary.

**Materials and Methods:**

We evaluated the effect of the smart antisnore pillow (SAP) in patients with OSAS in a prospective, noncontrolled, nonrandomized, pilot study. According to the apnea-hypopnea index (AHI), they were divided into two groups: mild-to-moderate OSAS group and severe OSAS group. Single-night polysomnography (PSG) with application of a SAP was performed. Thirty patients, 15 males and 15 females, 33–82 years old (mean age, 59.3 ± 12.9 years), completed the smart antisnore pillow therapy test. Among them, 23 patients had mild-to-moderate OSAS.

**Results:**

The SAP significantly improved the snore number (*p* = 0.018), snore index (*p* = 0.013), oxygen denaturation index (*p* = 0.001), total AHI (*p* = 0.002), and supine AHI (*p* = 0.002) in the mild-to-moderate OSAS group, but there was no significant improvement in the severe OSAS group.

**Conclusions:**

We concluded that the SAP is an effective positional therapy device for patients with OSAS of mild-to-moderate severity.

## 1. Introduction

Obstructive sleep apnea syndrome (OSAS) is characterized by snoring, sleep-related breathing pause, and daytime sleepiness [1] and is a result of a partial or complete collapse of the upper airways during sleep [2]. Patients with OSAS have an increased risk for hypertension, stroke, dementia, cardiac arrhythmias, and motor vehicle accidents [3].

The common treatment options for OSAS include continuous positive airway pressure (CPAP), upper airway surgery, and oral appliance use. Positional therapy (PT) can also be used, which includes methods for preventing patients with OSAS from sleeping in the worst sleeping position, usually the supine position. PT is regarded as an effective secondary therapy for OSAS in the American Academy of Sleep Medicine (AASM) practice guidelines [4]. It has been found to have a significant influence on snoring, OSAS severity, and apnea-hypopnea index (AHI). PT has a potential value in position-dependent snoring and OSAS treatment [5].

There are many devices used to prevent patients with OSAS from sleeping in the supine position by strapping an object, such as a ball or a vest, on their back [6–8]. However, these devices are redundant during sleep for most people and might cause discomfort, resulting in a poor long-term compliance [7, 9]. Pillows are habitual devices used during sleep to keep the head in a comfortable position. However, limited studies have been published on the effect of positional pillows for reducing the AHI and/or OSAS [10–12], and these pillows were usually made with a special shape, enabling neck extension [10, 11] or maintaining a person's head in the lateral position [12, 13].

Smart antisnoring pillows (SAP) are innovative devices that have an ordinary pillow shape, enabling sleeping in a natural position. These devices contain a shift control assembly base and mobile foam. The SAP device can detect the sleeper's snore by its audio sensors which are situated in the SAP device's lateral portion. The mobile foam can shift horizontally back and forth automatically after detecting a person's snoring sound, thereby changing their head and/or neck position. The mobile foam movement will stop until the snoring is undetected. The aim of this study was to evaluate the efficacy of SAP devices in the treatment of patients with OSAS.

## 2. Methods

### 2.1. Protocol Design and Participants

This was a single-center, single-treatment, noncontrolled, nonrandomized study. The patients were recruited from the population of the Chang Bing Show Chwan Memorial Hospital Sleep Center. The inclusion criteria were as follows: (1) age > 18 years, (2) clinical history of OSAS (snoring or breathing pause at sleep, daytime sleepiness) over 6 months, and (3) overnight baseline polysomnography (PSG) confirming an AHI ≥ 5 events/hour. The exclusion criteria included (1) serious medical or psychiatric diseases, such as heart failure, stroke, or chronic respiratory disorders, and (2) neck or shoulder problems preventing sleeping in a lateral position or turning the head. The eligible patients underwent a second, experimental, overnight PSG with the SAP device within 2 months after the baseline PSG.

This study was approved by the Institutional Review Board of Show Chwan Memorial Hospital. Informed consent was obtained from all patients before enrolment in this study. This study was performed in compliance with the Declaration of Helsinki.

### 2.2. Measurement of Sleep Quality

Sleep quality in this study was measured using two sleep questionnaires: the Pittsburgh Sleep Quality Index (PSQI) and the Epworth Sleepiness Scale (ESS). Patients completed these questionnaires prior to the baseline PSG.

### 2.3. PSG

PSG recording was performed in the Chang Bing Show Chwan Memorial Hospital Sleep Center Laboratory, which was accredited by the Taiwan Society of Sleep Medicine, using a digital polygraph system (Embla N7000, Broomfield, CO, USA) while the patients were breathing room air. Snoring was recorded using a piezo snore sensor positioned at the neck, over the larynx. A body position sensor was attached on the patients' anterior chest to define the posture, which was defined as supine and nonsupine (including right side, left side, or prone). Synchronized digital video recordings were also obtained on all patients and reviewed during the scoring process to confirm the body position. The sleep stage and obstructive respiratory events were scored according to the 2007 AASM manual. Snoring events were confirmed after deleting the abnormal spike wave. The noise caused by the SAP motion and operating shift control assembly was small and would not cause PSG snoring sensor recording errors. Obstructive apnea was defined as a 90% reduction in oronasal airflow for at least 10 seconds with continued ribcage and/or abdominal excursions. Hypopnea was defined as a 30% reduction in the airflow for at least 10 seconds with >3% oxygen desaturation. AHI was defined as the mean number of obstructive apnea and hypopnea events per hour of sleep.

### 2.4. Description of the SAP Device

**Figure 1:**
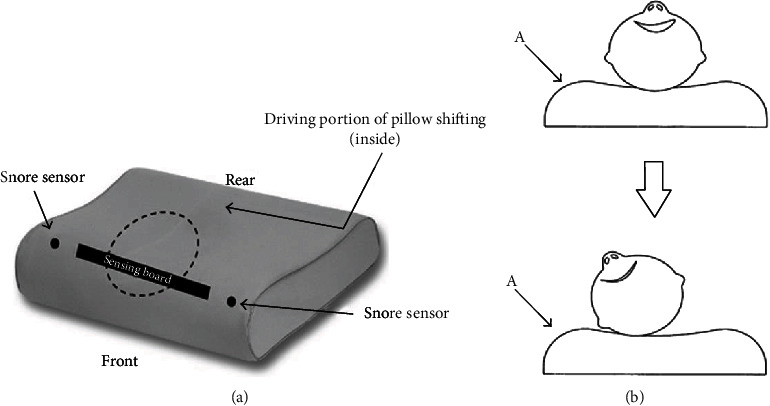
(a) Diagram of the smart antisnore pillow (SAP) device. (b) The working diagram of SAP: the head changes position when the snore sensors detect snoring and the SAP mobile foam shifts horizontally (A: snore sensor; ⟶: points out A's location).

The SAP device (US Patent No. US7676870 B2) is 50 cm × 30 cm × 11.5 cm in size and shaped as a usual pillow. It is composed of a shift control assembly base and a mobile seat foam. Two audio sensors that detect a person's snoring sound are situated in the lateral portion of the pillow (Figure 1(a)). When a snoring sound is detected, the shift control assembly is automatically activated to induce movement in the mobile seat. The mobile foam shifts horizontally back and forth, thus achieving movement of the head and/or neck, until the snoring ceases or the snore volume is undetectable (Figure 1(b)). In the present study, the SAP was set to trigger motion after detecting four consecutive snoring sounds.

### 2.5. Statistical Analysis

Continuous data were expressed as mean ± standard deviation, and categorical data were expressed as numbers with percentages. Based on the baseline AHI, patients were divided into two groups: mild-to-moderate OSAS group (5 ≤ AHI ≤ 30) and severe OSAS group (AHI > 30). The paired *t*-test or Wilcoxon's signed-rank test was used to compare the differences in the snoring number, snoring index, and AHI between the baseline and SAP therapy PSG. A two-tailed *p* value < 0.05 was considered statistically significant. Data analysis was performed using the statistical package IBM SPSS Statistics for Windows, version 24.0 (IBM Corp., Armonk, NY, USA).

## 3. Results

### 3.1. Demographic and Clinical Characteristics

Thirty patients, 15 males and 15 females, completed baseline PSG and SAP therapy PSG. All patients tolerated the experimental SAP treatment well. The mean age was 59.3 ± 12.9 (range, 33–82) years, the mean body mass index (BMI) was 27.6 ± 3.6 (range, 21.5–38.9) kg/m^2^, and the mean neck circumference was 37.03 ± 3.32 cm. The mean ESS and PSQI scores were 7.5 ± 3.5 and 9.10 ± 3.93, respectively. The patients' demographic characteristics are summarized in Table 1.

### 3.2. PSG

**Figure 2:**
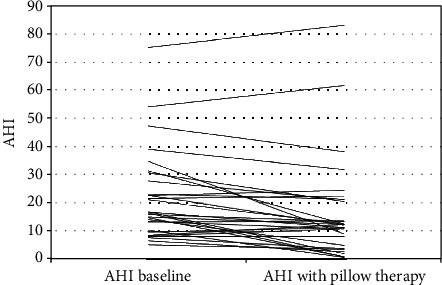
The individual effect of SAP therapy on the apnea-hypopnea index in 30 patients with obstructive sleep apnea.

The baseline AHI for all patients was in the range from 5.2 to 75.1 events/hour. There were 23 patients with mild-to-moderate OSAS and seven with severe OSAS. The SAP therapy AHI was in the range from 0.7 to 82.2 events/hour. The total AHI decreased in 22 patients (73%) and increased in 8 patients (27%) with the SAP therapy. The individual baseline and SAP therapy AHIs are shown in Figure 2.

The characteristics of the baseline and SAP therapy PSG for all patients are shown in Table 2. The SAP significantly decreased the snore number and snore index from 2406.7 ± 1173.5 to 1693.8 ± 1071.4 events (*p* = 0.004) and from 501.5 ± 235.1 to 360.9 ± 218.1 events/hour (*p* = 0.003), respectively. The oxygen desaturation index (ODI) decreased from 15.8 ± 16.3 to 7.8 ± 2.5 (*p* = 0.007), but the average oxygen saturation did not show obvious change (*p* = 0.322). The mean AHI also significantly decreased from 21.8 ± 15.7 to 16.5 ± 17.8 events/hour (*p* = 0.001). In particular, the supine AHI was significantly decreased from 27.3 ± 17.5 to 20.4 ± 19.9 events/hour (*p* = 0.005), but the SAP has no significant effect on the nonsupine AHI (*p* = 0.984). Although stage N1 increased from 36.9 ± 20.1 to 45.9 ± 24.0 (*p* = 0.037) after SAP therapy, there was no obvious change in sleep efficiency, stages N2 and N3, and REM stage.

Comparison of the significant PSG variables, snoring number, snore index, and total and supine AHI between the baseline and SAP therapy PSG in the mild-to-moderate OSAS and severe OSAS groups is presented in Table 3. The SAP had a significant effect in decreasing the ODI, snoring number, snore index, and total and supine AHI in the mild-to-moderate OSAS group but had no significant effect in the severe OSAS group. Furthermore, the SAP had no significant effect on the average oxygen saturation and nonsupine AHI in both groups.

## 4. Discussion

CPAP is the gold standard treatment for OSAS [14]. Untreated OSAS increases the risk of fatal cardiovascular events [15]. In addition, those patients are also associated with significant psychosocial consequences, such as decreased quality of life, impaired cognitive function, and increased depressive symptoms [16]. Despite the known risks of untreated OSAS and documented benefits of CPAP, nonadherence to therapy is a major issue. The CPAP adherence rates are variable; in the Asian populations, the rates are 38–90% [17–24], whereas in the Western populations, there is a 37.3–87.5% adherence rate [25–30]. There are many factors affecting the CPAP adherence in patients with OSAS, including age, comorbidities, ESS score, AHI, treatment titration procedures, device factors (cost, inconvenience, and discomfort), and psychological and social factors [14, 19, 20, 24]. Thus, PT or alternative therapies are valuable for patients with OSAS with nonadherence to CPAP.

In this study, the SAP device could reduce the total and supine AHI, snore number, and snore index in patients with mild-to-moderate OSAS. To the best of our knowledge, SAP is the first mobile positional therapy device to alter a person's head and/or neck position without changing the trunk position. At the same time, the arousal index did not increase during SAP therapy. The traditional PT included preventing patients with OSAS from sleeping in the supine position and keeping the trunk in a lateral position [31]. However, some studies showed that the head and/or neck position can affect the upper airway with the trunk in the supine position. Head posture had a marked effect on the collapsibility and site of collapse of the passive upper airway by anaesthesia [32]. The sniffing position with neck extension could increase the oropharyngeal airway size to maximum and decrease the closing pressures of the oropharynx and velopharynx in paralyzed patients with OSAS [33]. In a drug-induced sleep endoscopic observation, head rotation improved the upper airway collapse in supine-sleeping patients with OSAS [34]. van Kesteran and colleagues used two position sensors to detect the head and body position simultaneously during sleep. Their data showed that in 46.2% of the trunk supine position-dependent group, the head position considerably influenced the AHI (AHI was >5 higher when the head was also in a supine position compared to when the head was turned to the side). AHI might be alleviated through rotation of the head sideways while the trunk remains in a supine position [35]. In our study, the SAP device could reduce the supine AHI in patients with OSAS, indicating that the SAP-induced change in the head and neck position could open the collapsed upper airway even when the truck remained in a supine position. This result was similar to that of van Kesteran et al.'s investigation. The SAP had no significant improvement effect on the nonsupine AHI. These results implied that the SAP motion would not change the head and neck position significantly when a person's trunk is in a lateral and prone position.

The ODI is the average number of times per hour that oxygen saturation decreases per hour. It has a stronger correlation and is a better predictor for AHI in patients with OSAS [36]. In our study, the SAP device also improved the parameter in patients with mild-to-moderate OSAS. The ODI was associated with hypertension [37]. Further studies should be designed for the long-term usage of the SAP device.

Our study has some limitations. First, we recognize the limited number of patients in this study. Second, the single-night baseline and SAP therapy PSG might have induced first-night effect bias. Third, we did not follow-up the AHI and adherence after long-term usage of SAP therapy.

## 5. Conclusions

In conclusion, the SAP is an effective positional therapy device that can improve the total and supine AHI, snore number, and snore index in patients with mild-to-moderate OSAS by shifting their head and neck position. However, this device had no significant effect in patients with severe OSAS. Future studies should be directed towards understanding the oropharyngeal anatomic change during SAP activity, its long-term effect, neck comfort, and patients' compliance.

## Figures and Tables

**Table 1:** Demographic characteristics of 30 patients.

Variable	Numbers/mean ± SD
Female/male	15/15
Age (years)	59.30 ± 12.93
BMI (kg/m^2^)	27.35 ± 3.62
ESS	7.53 ± 3.53
PSQI	9.10 ± 3.93
Neck circumference (cm)	37.03 ± 3.32

Data are given as numbers or mean ± SD. BMI: body mass index; ESS: Epworth Sleepiness Scale; PSQI: Pittsburgh Sleep Quality Index.

**Table 2:** Polysomnographic variables of baseline and SAP therapy of 30 patients.

Variable	Baseline	SAP therapy	*p* value
Sleep efficiency (%)	77.8 ± 12.1	75.7 ± 13.8	0.473
Stage N1 (%)	36.9 ± 20.1	45.9 ± 24.0	0.037
Stage N2 (%)	49.0 ± 20.6	43.1 ± 22.1	0.157
Stage N3 (%)	1.1 ± 3.0	0.2 ± 0.4	0.132
REM (%)	13.0 ± 7.7	10.8 ± 5.3	0.086
Supine body position (%)	74.3 ± 23.7	73.1 ± 24.8	0.783
Nonsupine body position (%)	25.7 ± 23.7	26.9 ± 24.8	0.781
Arousal index (events/hour)	30.0 ± 19.7	32.91 ± 13.2	0.346
Average oxygen saturation (%)	91.0 ± 17.3	94.2 ± 2.3	0.322
ODI (events/hour)	15.8 ± 16.3	7.8 ± 2.5	0.007
Snore number	2406.7 ± 1173.5	1693.8 ± 1071.4	0.004
Snore index (events/hour)	501.5 ± 235.1	360.9 ± 218.1	0.003
AHI (events/hour)	21.8 ± 15.7	16.5 ± 17.8	0.001
Supine AHI (events/hour)	27.3 ± 17.5	20.4 ± 19.9	0.005
Nonsupine AHI (events/hour)	4.0 ± 6.3	4.1 ± 9.3	0.984

Data are given as mean ± SD. SAP: smart antisnore pillow; REM: rapid eye movement; ODI: oxygen denaturation index; AHI: apnea-hypopnea index.

**Table 3:** The effects of SAP in different baseline AHI severity categories.

Numbers	5 ≤ AHI ≤ 30			AHI > 30		
	23			7		
Variable	Baseline	SAP	*p* value	Baseline	SAP	*p* value
Average oxygen saturation (%)	94.5 ± 1.6	95.9 ± 1.3	0.072	92.5 ± 3.1	92.4 ± 3.4	0.958
ODI (events/hour)	10.3 ± 1.9	2.9 ± 2.6	0.001	28.5 ± 19.4	14.6 ± 8.7	0.244
Snore number	2528.0 ± 1231.3	1786.6 ± 1050.7	0.018	2007.9 ± 924.5	1388.7 ± 1165.5	0.063
Snore index (events/hour)	524.8 ± 247.9	377.0 ± 218.7	0.013	425.0 ± 181.2	307.9 ± 224.2	0.176
AHI (events/hour)	14.8 ± 6.2	10.3 ± 7.2	0.002	44.6 ± 15.9	36.6 ± 27.0	0.128
Supine AHI (events/hour)	21.4 ± 13.5	14.5 ± 12.2	0.002	46.9 ± 15.0	40.0 ± 28.1	0.398
Nonsupine AHI (events/hour)	14.7 ± 11.5	11.8 ± 15.0	0.401	24.3 ± 20.7	19.3 ± 12.7	0.655

Data are given as mean ± SD. SAP: smart antisnore pillow; ODI: oxygen denaturation index; AHI: apnea-hypopnea index.

## Data Availability

Data measured or analysed during this study are available from the corresponding author on request.
